# Life engagement in people living with schizophrenia: predictors and correlates of patient life engagement in a large sample of people living in the community

**DOI:** 10.1017/S0033291723002106

**Published:** 2023-12

**Authors:** Antonio Vita, Stefano Barlati, Giacomo Deste, Gabriele Nibbio, David L. Penn, Amy E. Pinkham, Roger S. McIntyre, Philip D. Harvey

**Affiliations:** 1 Department of Clinical and Experimental Sciences, University of Brescia, Brescia, Italy; 2 Department of Mental Health and Addiction Services, ASST Spedali Civili of Brescia, Brescia, Italy; 3 Department of Psychology, University of North Carolina, Chapel Hill, NC, USA; 4 School of Psychology, Australian Catholic University, Melbourne, VIC, Australia; 5 Department of Psychology, School of Behavioral and Brain Sciences, The University of Texas at Dallas, Richardson, TX, USA; 6 Department of Psychiatry and Pharmacology, University of Toronto, Toronto, Canada; 7 Brain and Discovery Foundation (BCDF), Toronto, Canada; 8 Department of Psychiatry and Behavioral Sciences, University of Miami Miller School of Medicine, Miami, FL, USA; 9 Research Service, Miami VA Healthcare System, Miami, FL, USA

**Keywords:** Integrated intervention, life engagement, patient-reported outcome, psychosocial functioning, recovery, schizophrenia, wellness

## Abstract

**Background:**

Life engagement represents a holistic concept that encompasses outcomes reflecting life-fulfilment, well-being and participation in valued and meaningful activities, which is recently gaining attention and scientific interest. Despite its conceptual importance and its relevance, life engagement represents a largely unexplored domain in schizophrenia. The aims of the present study were to independently assess correlates and predictors of patient life engagement in a large and well-characterized sample of schizophrenia patients.

**Methods:**

To assess the impact of different demographic, clinical, cognitive and functional parameters on life engagement in a large sample of patients with schizophrenia, data from the social cognition psychometric evaluation project were analyzed.

**Results:**

Overall schizophrenia and depressive symptom severity, premorbid IQ, neurocognitive performance, social cognition performance both in the emotion processing and theory of mind domains, functional capacity, social skills performance and real-world functioning in different areas all emerged as correlates of patient life engagement. Greater symptom severity and greater impairment in real-world interpersonal relationships, social skills, functional capacity and work outcomes emerged as individual predictors of greater limitations in life engagement.

**Conclusions:**

Life engagement in people living with schizophrenia represents a holistic and complex construct, with several different clinical, cognitive and functional correlates. These features represent potential treatment targets to improve the clinical condition and also facilitate the process of recovery and the overall well-being of people living with schizophrenia.

## Introduction

### Background

Schizophrenia is a debilitating mental disorder, characterized by the presence of positive and negative symptoms, as well as impaired neurocognition and social cognition and deficits in social skills that contribute to poor functional outcomes (Galderisi et al., 2014; Harvey & Strassnig, 2012).

Life engagement represents a holistic concept that encompasses outcomes reflecting life-fulfilment, well-being and participation in valued and meaningful activities, which is recently gaining great attention and scientific interest (Bartrés-Faz, Cattaneo, Solana, Tormos, & Pascual-Leone, 2018). It includes positive health aspects relating to both neurocognition and social cognition, as well as vitality, motivation and reward, and the ability to experience pleasure (McIntyre et al., 2022). Life engagement is an important patient-reported outcome, a category of parameters that takes into account the patient's subjective view and personal experiences. Such approaches allow for the development of more patient-centric interventions and treatments (Sartorius, 2014), and for these reasons, patient-reported outcomes are becoming increasingly prioritized in clinical trials (Kieffer, Miller, Chacko, & Robertson, 2020).

For instance, it can be frequently observed that people with schizophrenia living in the community do not feel that their occupations are worthwhile or important to them, or that their daily activities are valuable. This further complicates the difficulty they face in establishing and maintaining meaningful interpersonal relationships such as friendships and romantic relationships (Bonfils, Lysaker, Minor, & Salyers, 2019; Mucci et al., 2021).

They also often report very low levels of motivation in their lives, particularly as regards intrinsic motivation, and this has a significant negative impact not only on their daily functioning and real-life outcomes but also on the effectiveness of psychosocial interventions (Saperstein, Fiszdon, & Bell, 2011; Velligan, Kern, & Gold, 2006). In this context, even simple acts such as taking the bus, buying groceries, meeting other people and even caring for one's own hygiene can become difficult tasks. Finding and maintaining a job position also shows relevant issues (Reddy, Llerena, & Kern, 2016).

These are all issues that can be commonly observed in clinical practice, particularly in rehabilitation settings, and they become even more evident if patients are directly asked questions about their engagement in life.

Despite its conceptual importance and its relevance as a priority phenomenon for the person living with schizophrenia, life engagement represents a largely unexplored domain in people living with severe mental disorders. This deficiency may be due to the relatively recent development of the concept itself, and also to the lack of dedicated tools to measure the construct in the context of mental health broadly (McIntyre et al., 2022). In fact, a recent systematic review (McIntyre et al., 2022) demonstrates that despite the availability of a number of patient-reported outcome assessment instruments, only two validated tools directly explore patient life engagement, the Life Engagement Test (Scheier et al., 2006) and the Engaged Living Scale (Trompetter et al., 2013). However, these tools were not conceived and developed to be used in clinical contexts and are yet to be validated in clinical populations. Moreover, the foregoing scales do not fully assess the cognitive and vitality aspects underlying the concept of patient life engagement and were not designed to reflect longitudinal changes that a patient might experience following successful treatment (Correll, Ismail, McIntyre, Rafeyan, & Thase, 2022*a*). In this context, selected items from scales that are already well validated and widely used could provide important insight on life engagement in clinical contexts as well as in randomized trials (Baandrup, Rasmussen, Mainz, Videbech, & Kristensen, 2022).

The increasing interest in life engagement provided the impetus to develop a conceptual framework for patient life engagement specifically dedicated to people living with major depressive disorder (MDD) (Weiss et al., 2021) and to the development of a subscale of the Inventory of Depressive Symptomatology (Rush, Carmody, & Reimitz, 2000) to measure life engagement, which was clinically validated in separate studies (MacKenzie, Therrien, Brown, Weiss, & Meehan, 2021; Thase et al., 2019). With an approach similar to that used for MDD, a construct derived from the Positive and Negative Syndrome Scale (PANSS) (Kay, Fiszbein, & Opler, 1987) was developed through a modified Delphi process specifically to assess patient life engagement in people living with schizophrenia (Correll, Ismail, McIntyre, Rafeyan, & Thase, 2022*b*). Comprising 11 items and dubbed PANSS-11 or PANSS-Life Engagement, this construct was recently validated in a large sample of patients using data obtained from three pharmacological trials (Correll et al., 2015; Ismail et al., 2020*a*, 2020*b*; Kane et al., 2015; Laszlovszky et al., 2021; Marder et al., 2017).

Patient life engagement however remains a relatively unexplored domain in people living with schizophrenia, and a thorough examination of clinical, cognitive and functional parameters might influence this distal but important outcome could help to understand not only its determinants but also which factors might represent targets for treatment that could provide an even more meaningful improvement in patients’ lives (Correll et al., 2022*b*). Moreover, better defining the correlates of life engagement in the clinical context with validated and available instruments could further increase the possibility of characterizing each individual patient, which could eventually improve the ability to devise, implement and optimize personalized treatment programs (Maj et al., 2021).

Symptom severity could have an important role in impairing life engagement in people living with schizophrenia. Clinical remission is an essential first step in the rehabilitation processes (Vita & Barlati, 2018), and symptom exacerbations and resulting relapses represent one of the most important limiting factors of functional and personal recovery (Jääskeläinen et al., 2013; Maj et al., 2021; Taylor & Jauhar, 2019).

Depressive symptoms are another frequent feature among people living with schizophrenia, which represents an important determinant of negative outcomes, including suicide. These symptoms can also be a relevant source of secondary negative symptoms (Peralta & Cuesta, 2009) and are closely connected to the concept of life engagement. In fact, people living with schizophrenia often report that it is hard for them to find reasons for living and that their life does not appear to have a purpose: this might represent a relevant area of overlap between depressive symptoms and low levels of life engagement (Scheier et al., 2006).

Social cognition and social skills may also have an important role. Social cognition deficits represent another central feature of schizophrenia and represent one of the strongest predictors of greater impairments in psychosocial functioning and poorer real-world outcomes (Deste et al., 2020; Harvey, Deckler, Jarskog, Penn, & Pinkham, 2019; Silberstein, Pinkham, Penn, & Harvey, 2018). Meaningful relationships are an essential part of life fulfillment, and as being able to interact in social circumstances represents one of the factors conceptualized as essential in patient life engagement (McIntyre et al., 2022), assessing whether deficits in specific social cognition domains or in social skills actually has an impact on currently validated life engagement measures could confirm the robustness of these measures and of their underlying concepts. Moreover, it could help understand the proportion of participants for which different clinical and cognitive elements could contribute in limiting this important outcome.

Finally, psychosocial functioning represents one of the most distal outcomes in the treatment of schizophrenia, but is clearly one of the most relevant aspects to take into account when considering a person's overall well-being and recovery goals. In fact, functioning represents the ultimate target of structured rehabilitation programs, as well as evidence-based treatments in modern psychiatry (Galderisi et al., 2014; Harvey & Strassnig, 2012; Vita & Barlati, 2019). The dimension of psychosocial functioning, thus, constitutes one of the essential components of life engagement, independently from the clinical context, and therefore has to be taken into account when exploring the determinants of this outcome.

The aims of the present study were to independently assess correlates and predictors of patient life engagement in a large and well-characterized sample of people living with schizophrenia.

## Materials and methods

### Study design and participants

To appropriately assess the impact of different clinical, cognitive and functional parameters on patient life engagement in a large sample of people living with schizophrenia, data from the social cognition psychometric evaluation (SCOPE) project (Pinkham et al., 2014) were analyzed.

For the present study, data from two different datasets of the five-phase project were taken into account and merged into a single database. The first dataset included data gathered from the third phase of the project, SCOPE phase 3 (Pinkham, Penn, Green, & Harvey, 2016), while data included from the second dataset were gathered for the fifth and final part of the project, SCOPE phase 5 (Pinkham, Harvey, & Penn, 2018). For the SCOPE 3 study, a total of 179 participants were recruited at two sites, the Southern Methodist University and the Miami Miller School of Medicine, while the SCOPE 5 study included a total of 218 participants recruited at three sites, the University of Texas at Dallas, the University of Miami Miller School of Medicine and the University of North Carolina at Chapel Hill.

Inclusion and exclusion criteria were identical in the two studies. Inclusion criteria were (I) diagnosis of schizophrenia or schizoaffective disorder, according to the Diagnostic and Statistical Manual of Mental Disorders, Fourth Edition (DSM-IV) (American Psychiatric Association, 1994), confirmed with the Mini International Neuropsychiatric Interview (Sheehan et al., 1998) and the Structured Clinical Interview for DSM Disorders Psychosis Module (First, Spitzer, Gibbon, & Williams, 2002), (II) stable clinical condition, meaning that participants had no hospitalization occurring in the previous two months, no change in the medication regimen for a minimum of 6 weeks and no medication dosage change for a minimum of 2 weeks.

Exclusion criteria were (I) presence or history of pervasive developmental disorder, including autism spectrum disorder, or of mental retardation with an IQ < 70, as defined by the diagnostic criteria reported in the DSM-IV, (II) presence or history of any medical or neurological illness that could have a negative impact on the functioning of the central nervous system, including epilepsy and seizures, neoplasms of the central nervous system structures and inflammatory or autoimmune disorders affecting the central nervous system, (III) presence of visual or hearing impairment severe enough to limit the participation of the patients in the assessment, (IV) no or very limited proficiency with English language, (V) presence of substance abuse in the past month and (VI) presence of active substance dependence in past 6 months.

As a small number of participants were recruited in both phases of the study, SCOPE 5 data from repeating participants were removed from the final sample. Therefore, the overall sample size of the present study is smaller than the sum of the number of participants recruited in the two SCOPE studies.

### Measures

#### Life engagement

Patient life engagement was measured using the PANSS-Life Engagement Subscale, also known as the PANSS-11. This construct is obtained by summing 11 items of the original assessment tool (Kay et al., 1987) that are strongly related to the core concepts of life engagement related to issues experienced by people living with schizophrenia: N1 (‘blunted affect’), N2 (‘emotional withdrawal’), N3 (‘poor rapport’), N4 (‘social withdrawal’), N5 (‘difficulties in abstract thinking’), N6 (‘lack of spontaneity and flow of conversation’), G6 (‘depression’), and G7 (‘motor retardation’), G13 (‘disturbances of volition’) G15 (‘preoccupation’) and G16 (‘active social avoidance’). Higher scores represent worse engagement in life. This construct was validated in a large sample of patients, totaling 1378 participants (Ismail et al., 2020*a*, 2020*b*; Laszlovszky et al., 2021), using data obtained from three different pharmacological trials (Correll et al., 2015; Kane et al., 2015; Marder et al., 2017), and currently represent the only clinically validated measure of patient life engagement specifically devised to assess this parameter in people living with schizophrenia.

#### Symptom severity

Global schizophrenia symptom severity was measured using the PANSS scale (Kay et al., 1987). To avoid potential collinearity with the PANSS-Life Engagement Subscale in the linear regression models, a construct composed by all items of the PANSS not included in the PANSS-Life Engagement Subscale, dubbed PANSSminusLE, was included in the main analyses.

Depressive symptoms were measured using the second edition of the self-report Beck Depression Inventory (BDI-II) (Beck, Steer, & Brown, 1987). The BDI-II represents a subjective measure of depressive symptoms, which might be more closely correlated to the patient-centric outcome of life engagement and better represent the patient's perspective in this context (Trivedi, Papakostas, Jackson, & Rafeyan, 2020; Weldring & Smith, 2013).

#### Social cognition

As social cognition represented the main focus of the SCOPE project, several measures of social cognitive performance were available in the present study. Only the measures that were included in both SCOPE phase 3 and phase 5 were included in the analyses.

For the Emotion Recognition domain, the Bell Lysaker Emotion Recognition Task (BLERT) (Bryson, Bell, & Lysaker, 1997) and the Penn Emotion Recognition Test (ER-40) (Kohler et al., 2003) were used. The total number of correctly identified emotions represents the final score in both tests.

For the Mental State Attribution/Theory of Mind domain, the Reading the Mind in the Eyes Test (EYES) (Baron-Cohen, Wheelwright, Hill, Raste, & Plumb, 2001), the Hinting Task (HINTING) (Corcoran, Mercer, & Frith, 1995) and The Awareness of Social Inferences Test, Part 3 (TASIT) (McDonald, Flanagan, Rollins, & Kinch, 2003) were used. All three tasks are scored as total number correct.

#### Global cognition

Neurocognitive performance was assessed with the tests from the MATRICS Consensus Cognitive Battery (Nuechterlein et al., 2008): Trail Making Test-Part A; Brief Assessment of Cognition in Schizophrenia (BACS) Symbol Coding; BACS Category Fluency (Animal Naming); Letter–Number Span and Hopkins Verbal Learning Test-Revised. These tests assess the cognitive domains that appear to be prominently impaired in people living with schizophrenia, such as processing speed, working memory and verbal memory (Nuechterlein et al., 2004). Following the recommendation of the developers of the battery, a global composite score was calculated by averaging the *t*-scores of all tests (NEUROCOG). Participants’ premorbid IQ was assessed using the Wide Range Achievement Test-3 Reading Subscale (WRAT-3) (Weickert et al., 2000).

#### Psychosocial functioning

The SCOPE project included several measures related to psychosocial functioning.

Functional capacity was assessed using the University of California San Diego (UCSD) Performance-Based Skills Assessment, Brief form (UPSA-B) (Mausbach, Harvey, Goldman, Jeste, & Patterson, 2007), a task that evaluates financial and communication skills involved in community contexts.

Social competence was assessed using the social skills performance assessment (SSPA) (Patterson, Moscona, McKibbin, Davidson, & Jeste, 2001), a role-playing task in which participants have to start and maintain a conversation in two different social situations. Interest, fluency, clarity, focus, overall abilities, social appropriateness and also negotiation ability and persistence are evaluated by a gold-standard rater.

Real-world functional outcomes were assessed with the informant-rated version of the Specific Level of Functioning Scale (SLOF) (Schneider & Struening, 1983). The interpersonal relationships, social acceptability, participation in daily activities and work skills subscales were included in the assessment battery. The SLOF represents one of the most reliable and valid instruments to assess real-world functioning in people living with schizophrenia, showing good construct validity and internal consistency in different large-scale studies (Harvey et al., 2011; Mucci et al., 2014).

Higher scores represent better psychosocial functioning for all included assessment tools.

#### Statistical analysis

To assess potential predictors of patient life engagement, correlation analyses between PANSS-Life Engagement and all continuous socio-demographic, clinical, cognitive and functional measures were performed. Independent samples *t* tests were used to assess potential gender differences in life engagement. As these variable selection analyses were conducted to identify potential predictors for regression models, no correction for multiple comparisons was applied (Heinze, Wallisch, & Dunkler, 2018).

To identify individual predictors of patient life engagement, multivariate linear regression analyses were performed, including potential predictors that emerged as correlated in univariate analyses. Multiple linear regressions were conducted using a stepwise procedure to assess which factors explained the largest proportion of the observed life engagement variance.

To assess the role of different cognition tasks and domains individually and separately, an additional regression analysis was conducted using only cognitive measures as potential predictors.

Collinearity between individual predictors was considered significant, according to conservative estimates, if the variance inflation factor (VIF) exceeded a value 4.0 or if tolerance was found to be below 0.25 (Alin, 2010).

As the number of potential predictors in each model was lower than one for every 20 observed subjects, the number of the included predictors was considered appropriate (Austin & Steyerberg, 2015; Schmidt, 1971).

Statistical analyses were performed using spss 15.0 software. Scatter plots were designed with JASP 0.16.4. *p*-values <0.05 (two-tailed) were considered significant.

## Results

### Correlation analyses

The characteristics of the sample are reported in Table 1.

**Table 1.** Characteristics of the sample

Variable	*N*, mean ± s.d.
*N*	361
M:F	244:117
Age	41.7 ± 12.0
Education years	12.9 ± 2.3
WRAT-3 (premorbid IQ)	94.4 ± 15.2
PANSS-Life Engagement	22.2 ± 7.42
PANSSminusLE (global symptoms severity)	39.7 ± 10.5
BDI-II (depressive symptoms)	15.5 ± 12.3
BLERT (emotion recognition)	13.5 ± 4.0
ER-40 (emotion recognition)	30.2 ± 5.2
EYES (theory of mind)	20.7 ± 5.5
HINTING (theory of mind)	13.4 ± 3.8
TASIT (theory of mind)	44.6 ± 7.6
NEUROCOG (global neurocognitive performance)	38.3 ± 7.2
UPSA-B (functional capacity)	69.8 ± 14.2
SSPA (social skills)	4.1 ± 0.5
SLOF interpersonal relationships	3.3 ± 0.9
SLOF social acceptability	4.5 ± 0.5
SLOF activities	4.5 ± 0.8
SLOF work	3.7 ± 0.8

BDI, Beck Depression Inventory; BLERT, Bell Lysaker Emotion Recogniton Task; ER-40, Penn Emotion Recognition Test; EYES, Reading the Mind in the Eyes; HINTING, Hinting Task; NEUROCOG, Global Cognitive Composite Score (*t*-score); PANSS, Positive and Negative Syndrome Scale; SLOF, Specific Level Of Functioning; SSPA, Social Skills Performance Assessment; TASIT, The Awareness of Social Inferences Task; UPSA-B, UCSD Performance-Based Skills Assessment, Brief; WRAT-3, Wide Range Achievement Test-3 Reading Subscale.

Correlation analyses are shown in Table 2. Premorbid lower IQ as measured by the WRAT-3 (*p* = 0.013), more severe symptoms as measured by PANSSminusLE (*p* < 0.001), more severe self-reported depressive symptoms as measured by the BDI-II (*p* < 0.001), poorer emotion recognition performance as measured by the ER-40 (*p* = 0.004), poorer theory of mind/mental state attribution performance as measured by the EYES (*p* = 0.018), HINTING (*p* = 0.001), and TASIT (*p* < 0.001) tasks, poorer global neurocognitive performance (NEUROCOG, *p* < 0.001), poorer functional capacity as measured by the UPSA-B (*p* < 0.001), poorer social skills as measured by the SSPA (*p* < 0.001) and poorer real work outcomes as measured by the interpersonal relationships (*p* < 0.001), activities (*p* < 0.001) and work (*p* < 0.001) subscales of the SLOF emerged as potential predictors of lower patient life engagement.

**Table 2.** Exploratory univariate analyses: correlations with life engagement

Variable	Pearson's *r*	*p*-Value
Age	−0.046	0.384
Education years	−0.055	0.294
WRAT-3 (premorbid IQ)	−0.131	0.013
PANSSminusLE (global symptoms severity)	0.442	<0.001
BDI-II (depressive symptoms)	0.207	<0.001
BLERT (emotion recognition)	−0.097	0.065
ER-40 (emotion recognition)	−0.151	0.004
EYES (theory of mind)	−0.125	0.018
HINTING (theory of mind)	−0.175	0.001
TASIT (theory of mind)	−0.236	<0.001
NEUROCOG (global neurocognitive performance)	−0.214	<0.001
UPSA-B (functional capacity)	−0.293	<0.001
SSPA (social skills)	−0.339	<0.001
SLOF interpersonal relationships	−0.483	<0.001
SLOF social acceptability	−0.076	0.150
SLOF activities	−0.139	0.009
SLOF work	−0.198	<0.001

BDI, Beck Depression Inventory; BLERT, Bell Lysaker Emotion Recogniton Task; ER-40, Penn Emotion Recognition Test; EYES, Reading the Mind in the Eyes; HINTING, Hinting Task; LE, Life Engagement; NEUROCOG, Global Cognitive Composite Score (*t*-score); PANSS, Positive and Negative Syndrome Scale; SLOF, Specific Level Of Functioning; SSPA, Social Skills Performance Assessment; TASIT, The Awareness of Social Inferences Task; UPSA-B, UCSD Performance-Based Skills Assessment, Brief; WRAT-3, Wide Range Achievement Test-3 Reading Subscale.

Scatter plots for significant correlation analyses are reported in the online Supplementary Materials.

### Regression analyses

Results of the main multivariate regression model are reported in Table 3.

**Table 3.** Predictors of life engagement: global linear regression model

Dependent variable	Individual predictors	Standardized beta	*t*	*p*-Value	VIF	Step adj. *R*^2^
	SLOF interpersonal relationships	−0.375	−7.582	<0.001	1.349	0.220
	PANSSminusLE (global symptoms severity)	0.318	7.177	<0.001	1.082	0.333
	SSPA (social skills)	−0.170	−3.550	<0.001	1.261	0.371
	UPSA-B (functional capacity)	−0.162	−3.405	0.001	1.242	0.386
	SLOF work	−0.111	−2.203	0.028	1.386	0.393
	Model: *F* = 44.269, *R*^2^ = 0.402, adj. *R*^2^ = 0.393, *p* < 0.001					

Potential predictors: WRAT-3 (premorbid IQ), PANSSminusLE (global symptoms severity), BDI-II (depressive symptoms), ER-40 (emotion recognition), EYES (theory of mind), HINTING (theory of mind), TASIT (theory of mind), NEUROCOG (global neurocognitive performance), UPSA-B (functional capacity), SSPA (social skills), SLOF interpersonal relationships, SLOF activities, SLOF work.

BDI, Beck Depression Inventory; ER-40, Penn Emotion Recognition Test; EYES, Reading the Mind in the Eyes; HINTING, Hinting Task; LE, Life Engagement; NEUROCOG, Global Cognitive Composite Score (*t*-score); PANSS, Positive and Negative Syndrome Scale; SLOF, Specific Level Of Functioning; SSPA, Social Skills Performance Assessment; TASIT, The Awareness of Social Inferences Task; UPSA-B, UCSD Performance-Based Skills Assessment, Brief; WRAT-3, Wide Range Achievement Test-3 Reading Subscale.

Greater impairment in real-world interpersonal relationships (*p* < 0.001), greater schizophrenia symptoms severity (*p* < 0.001), greater social skills deficits (*p* < 0.001), greater functional capacity impairment (*p* = 0.001) and greater impairment in real-world work outcomes (*p* = 0.028) emerged as individual predictors of poorer patient life engagement.

Considering the multiple regression analysis dedicated to cognitive variables, as shown in Table 4, poorer performance in the theory of mind/mental state attribution domain as measured by the TASIT (*p* = 0.001) and by the HINTING (*p* = 0.025) tasks emerged as individual predictors of poorer patient life engagement.

**Table 4.** Predictors of life engagement: cognitive predictors

Dependent variable	Individual predictors	Standardized beta	*t*	*p*-Value	VIF	Step adj. *R*^2^
Life engagement	TASIT (theory of mind)	−0.189	−3.338	0.001	1.171	0.053
	HINTING (theory of mind)	−0.127	−2.247	0.025	1.171	0.065
	Model: *F* = 12.830, *R*^2^ = 0.070, adj. *R*^2^ = 0.065, *p* < 0.001					

Potential predictors: ER-40 (emotion recognition), EYES (theory of mind), HINTING (theory of mind), TASIT (theory of mind), NEUROCOG (global neurocognitive performance).

ER-40, Penn Emotion Recognition Test; EYES, Reading the Mind in the Eyes; HINTING, Hinting Task; NEUROCOG, Global Cognitive Composite Score (*t*-score); TASIT, The Awareness of Social Inferences Task.

## Discussion

The present study aimed to assess correlates and predictors of patient life engagement among a wide array of demographic, clinical, cognitive and functional characteristics in a large sample of people diagnosed with schizophrenia living in the community. Global and depressive symptom severity, premorbid IQ, neurocognitive performance, social cognition performance both in the emotion processing and theory of mind domains, functional capacity, social skills performance and real-world functioning in different areas all emerged as correlates of patient life engagement. Greater symptoms severity and greater impairment in real-world interpersonal relationships, social skills, functional capacity and work outcomes emerged as individual predictors of greater limitations in patient life engagement.

People living with schizophrenia often report that their occupations are not worthwhile and that their daily activities are not important to them. They often struggle to find reasons to live, and purposes and meaning in their existence, and they often report that their struggles are unimportant to other people around them.

Our findings are in line with the theoretical concept of life engagement, which represents a complex and holistic combination of life-fulfilment, well-being and experience of value and meaning in life, and therefore encompasses, and is determined by, different aspects such as motivation, vitality and the ability to interact with others (Bartrés-Faz et al., 2018; McIntyre et al., 2022).

Many of these aspects are impaired in people living with schizophrenia and, as shown by the results of the present study, all contribute, to various extents, in determining poorer life engagement levels.

These results further validate the utility of the PANSS-Life Engagement Subscale as a reliable measure of patient life engagement, as the variance observed in its score was determined by a wide array of different factors, all relevant in the conceptual framework of life engagement. The PANSS-Life Engagement or PANSS-11 was already validated in previous work (Ismail et al., 2020*a*; Laszlovszky et al., 2021), but results of the present study suggest that it represents a valuable and easily implemented tool for the assessment of life engagement in people living with schizophrenia, both in clinical trials and in the clinical practice, particularly due to the lack of validated measures of life engagement, specifically designed for people with schizophrenia (McIntyre et al., 2022) and due to the diffusion of the use of the PANSS in different research and clinical contexts (Maj et al., 2021).

In this context, monitoring life engagement in clinical practice could represent a valuable asset in the assessment of the patient's rehabilitation process. While directly asking the patient about its engagement in life remains for the present time the optimal assessment method; the PANSS-11 could provide an objective and rapidly obtainable measure in clinical settings and represent a standardized and validated measure in clinical research.

Real-world interpersonal relationships emerged as an important and the strongest predictor of reduced patient life engagement in people living with schizophrenia. This is an interesting finding, supporting the close theoretical link between real-world social functioning and patient life engagement and strengthening the notion that a reduced social life, comprising feelings of loneliness, is one of the strongest determinants of reduced overall well-being also in the patients’ perspective (McIntyre et al., 2022; Park et al., 2020).

Focusing on real-world outcomes, particularly as regards interpersonal relationships, should therefore be always considered a priority in the treatment process of people living with schizophrenia. Encouraging the development of meaningful relationships and providing opportunities for socialization can represent a relevant part of rehabilitation programs, and monitoring progresses in this context should be considered an important step in the overall assessment of patients’ functioning, of the progress of patients’ rehabilitation and also of life engagement.

Symptom severity emerged as the most relevant predictor of life engagement after real-world interpersonal relationships. Clinical stability represents an essential prerequisite for the process of recovery (Vita & Barlati, 2018), and symptoms exacerbations and relapses represent indeed one of its main limiting factors (Jääskeläinen et al., 2013; Taylor & Jauhar, 2019). In this regard, optimizing pharmacological treatment (Leucht et al., 2021, 2017) and implementing dedicated evidence-based psychosocial interventions (Bighelli et al., 2021) could represent effective strategies not only with the aim of achieving symptoms remission and clinical stability but also to improve the process of recovery and, ultimately, patient life engagement.

Besides real-world interpersonal relationships, functional capacity and work outcomes also emerged as individual predictors in the linear regression model, further highlighting the importance of functional capacity as a mediator between clinical symptoms and overall disability (Harvey & Strassnig, 2012; Mucci et al., 2021) and the relationship between global psychosocial functioning and the holistic concept of patient life engagement (Correll et al., 2022*b*).

Several psychosocial interventions have been shown to provide significant improvements in real-world outcomes of people living with schizophrenia (Bighelli et al., 2021; Solmi et al., 2023; Vita et al., 2022*b*): cognitive remediation (Lejeune, Northrop, & Kurtz, 2021; Vita et al., 2021), social skills training (Turner et al., 2018), physical exercise (Stubbs et al., 2018), cognitive behavioral therapy for psychosis (Bighelli et al., 2018) and meta-cognitive training (Penney et al., 2022) all have solid meta-analytic evidence of effectiveness, not only in their main treatment targets but also in improving psychosocial functioning and real-world outcomes.

Considering the role of psychosocial functioning in patient life engagement, providing evidence-based treatments in mental health services could result in even more substantial benefits for people living with schizophrenia.

Social skills performance also positively contributed to patient life engagement. These findings suggest that social skills training (Turner et al., 2018) could exert a specific positive effect on life engagement: in fact, if sufficient resources are available, social skills training should be provided alongside cognitive remediation interventions, as combining evidence-based treatments provides substantially greater improvements in cognitive as well as functional outcomes (Lejeune et al., 2021; Nibbio et al., 2020; van Duin et al., 2019; Vita et al., 2023).

Depressive symptoms are frequently observed in schizophrenia, and they also appear to be related to life engagement. Depressive symptoms should be carefully assessed and receive dedicated treatment in people with schizophrenia (Baynes et al., 2000; Conley, Ascher-Svanum, Zhu, Faries, & Kinon, 2007; Lako et al., 2012; Mulholland & Cooper, 2000).

Better performance in both theory of mind and emotion recognition domains emerged as correlates of better life engagement. This was an expected result, as the ability to meaningfully interact with other people is a key component of life engagement, and social cognition is essential for valid interpersonal reactions (McIntyre et al., 2022). In the regression analysis dedicated specifically to cognitive variables, two different tasks measuring theory of mind/mental state attribution performance emerged as individual predictors, while no measure of emotion recognition emerged in this regression model. This result might be interpreted in light of the fact that theory of mind might be considered a more complex, higher-order domain than emotion recognition, linked to higher level inferential processing (Buck, Healey, Gagen, Roberts, & Penn, 2016), and therefore deficits in this area might have a stronger relationship with more distal outcomes such as patient life engagement. These findings confirm the importance of providing treatment for cognitive deficits for people living with schizophrenia: recent meta-analytic studies including large samples of participants show that cognitive remediation interventions are effective in improving social cognition (Vita et al., 2021), and several effective interventions specifically targeting social cognition are currently available (Yeo, Yoon, Lee, Kurtz, & Choi, 2022).

All these factors and all the available and targeted evidence-based treatments could provide substantial benefits to patients’ life engagement and, consequently, to their overall rehabilitation process. This could in turn have a positive impact on patients’ real-world functioning outcomes and even on their quality of life. In fact, feeling that their daily activities are worthwhile and that their rehabilitation goals are achievable and meaningful for their lives could represent an essential step to improve the motivation, the sense of worth and the overall well-being of people with schizophrenia living in the community.

This study presents a number of strengths. First, to the best of our knowledge, this is the first study to investigate correlates and predictors of life engagement in people living with schizophrenia, highlighting the importance of analyzing patient-reported outcomes and determinants. Moreover, the large sample size, the multicentric structure and the inclusive recruitment criteria of the SCOPE project allow assessment of a sample of individuals with schizophrenia, living in the community, that can be considered highly representative of the investigated population. This also allowed to avoid substantial issues of ranges restrictions in the correlation and regression analyses. Finally, the use of a wide array of well-validated assessment tools allows exploration of the role and the relative importance of several different categories of variables.

Some limitations also have to be taken into account. No assessment was performed regarding stability of patient life engagement and its changes over time were not explored: this is an intrinsic limitation related to the cross-sectional nature of the assessments, which will need to be overcome in future longitudinal studies. Finally, no data concerning pharmacological treatment were included in the present analyses: this is another issue that requires further investigation.

Future research should also focus on establishing the effectiveness of different pharmacological treatments and psychosocial interventions on life engagement in people living with schizophrenia, and on developing more direct and simple measures of patient life engagement for use in the clinical practice. Future interventional research could also include measures of life engagement in adults with schizophrenia as a relevant outcome measure, especially when adjudicating relative benefits of psychotropic agents in persons living with schizophrenia. Other correlates of life engagement that were not assessed in the present study, such as type and dosage of medications, autistic symptoms severity (Abu-Akel et al., 2022; Vita et al., 2020), internalized stigma (Barlati et al., 2022; Carrara & Ventura, 2018) or sleep disturbances (Chouinard, Poulin, Stip, & Godbout, 2004; Kaskie, Graziano, & Ferrarelli, 2017), should also be explored further in this population. Comorbidity, particularly with substance use disorders, could also negatively affect engagement: this could be of particular interest, as it could also represent a target of treatment (Bennett, Bradshaw, & Catalano, 2017; Hunt, Large, Cleary, Lai, & Saunders, 2018; Khokhar, Dwiel, Henricks, Doucette, & Green, 2018). Finally, cost-effectiveness studies should seek to determine whether improving life engagement reduces the overall cost of the illness and would warrant paying for the treatment.

In conclusion, the results of the present study show that many different factors represent correlates of life engagement in people living with schizophrenia, and this confirms the holistic and complex structure of this construct. Patient life engagement represents an important patient-reported outcome that requires further study both in clinical trials and in epidemiological research conducted on clinical populations.

## Supporting information

Vita et al. supplementary material Vita et al. supplementary material

## Supplementary material

The supplementary material for this article can be found at https://doi.org/10.1017/S0033291723002106
