# Supplementary material for: Life engagement in people living with schizophrenia: predictors and correlates of patient life engagement in a large sample of people living in the community
Source: Psychol Med. 2023 Jul 31;53(16):7943–52. doi: 10.1017/S0033291723002106 (PMC10755242; doi:10.1017/S0033291723002106)
Supplement: Vita et al. supplementary material [file S0033291723002106sup001.docx]

**Supplementary Materials:**

**Scatter plots for significant correlation analyses – Life Engagement**

**Clinical variables**


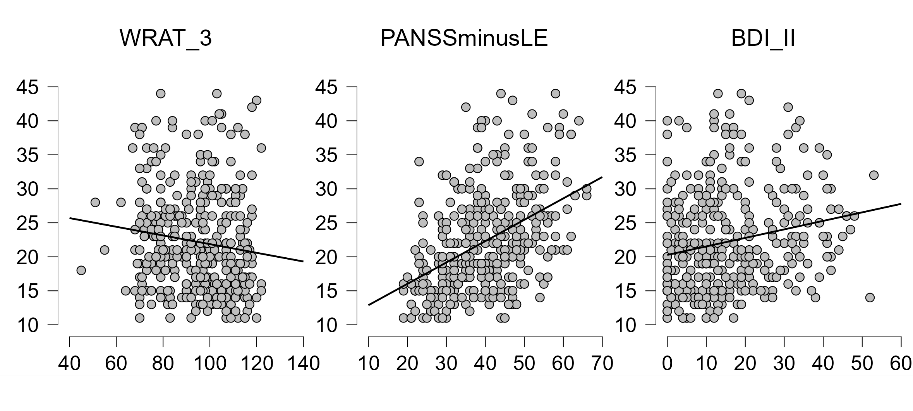


**Cognitive variables**


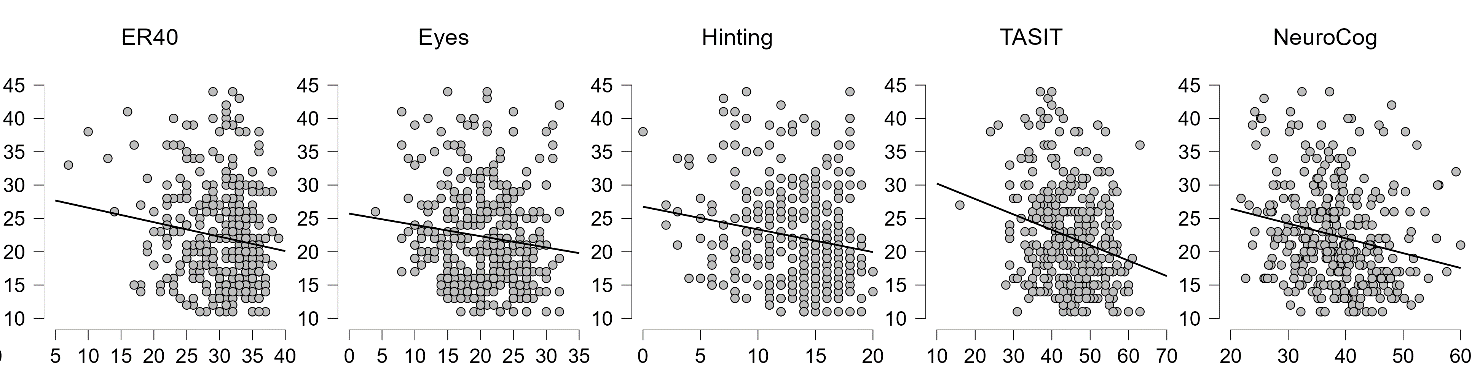


**Functional variables**


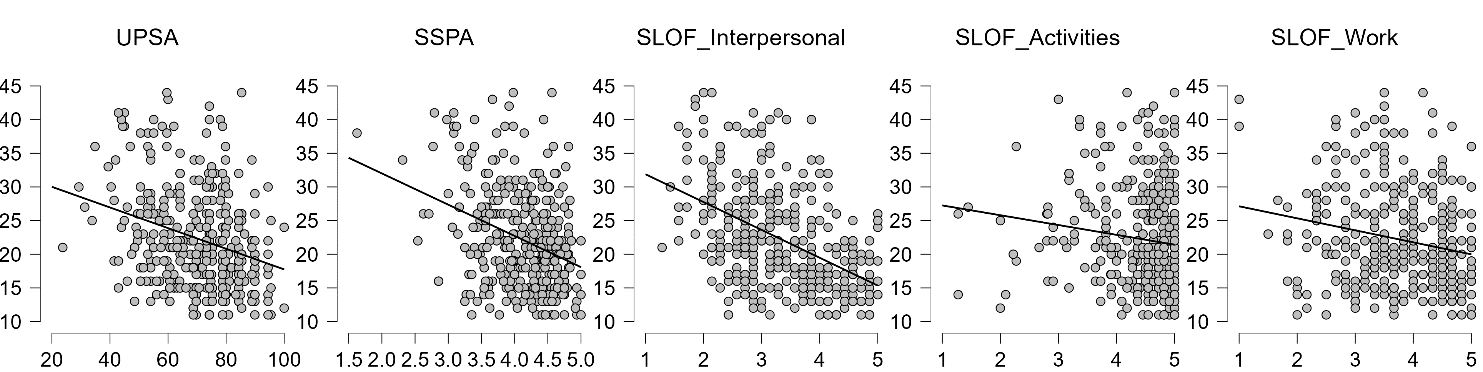


BDI-II: Beck Depression Inventory

ER40: Penn Emotion Recognition Test

Eyes: Reading the Mind in the Eyes

Hinting: Hinting Task

LE: Life Engagement

NeuroCog: Global Cognitive Composite Score (t-score)

PANSS: Positive and Negative Syndrome Scale

SLOF: Specific Level OF Functioning

SSPA: Social Skills Performance Assessment

TASIT: The Awareness of Social Inferences Task

UPSA: UCSD Performance-Based Skills Assessment

WRAT-3: Wide Range Achievement Test-3 Reading subscale
